# Using Therapeutic Drug Monitoring to Treat KPC-Producing *Klebsiella pneumoniae* Central Nervous System Infection With Ceftazidime/Avibactam

**DOI:** 10.1093/ofid/ofaa349

**Published:** 2020-08-18

**Authors:** Mohamad Yasmin, Jennifer Hanrahan, Steven Marshall, Thomas P Lodise, Liang Chen, Federico Perez, Barry Kreiswirth, Robert A Bonomo

**Affiliations:** 1 Research Service, Louis Stokes Cleveland Department of Veterans Affairs Medical Center, Cleveland, Ohio, USA; 2 Division of Infectious Diseases, University of Toledo College of Medicine and Life Sciences, Toledo, Ohio, USA; 3 Department of Pharmacy Practice, Albany College of Pharmacy and Health Sciences, Albany, New York, USA; 4 Center for Discovery and Innovation, Hackensack Meridian Health, Nutley, NJ, USA; 5 Department of Medical Sciences, Hackensack Meridian School of Medicine, Nutley, NJ, USA; 6 Department of Medicine, Case Western Reserve University, Cleveland, Ohio, USA; 7 Geriatric Research, Education and Clinical Center, Louis Stokes Cleveland VA Medical Center, Cleveland, Ohio, USA; 8 Departments of Molecular Biology & Microbiology, Pharmacology, Biochemistry, and Proteomics & Bioinformatics, Case Western Reserve University, Cleveland, Ohio, USA; 9 CWRU-Cleveland VAMC Center for Antimicrobial Resistance and Epidemiology (Case VA CARES), Cleveland, Ohio, USA

**Keywords:** antimicrobial resistance, ceftazidime/avibactam, CNS infections, KPC, therapeutic drug monitoring

## Abstract

This report describes the treatment of *Klebsiella pneumoniae* carbapenemase (KPC)–3–producing multidrug-resistant *K. pneumoniae* with ceftazidime/avibactam (CAZ-AVI) in a patient who developed postneurosurgical meningitis and bacteremia. Therapeutic drug monitoring of cerebrospinal fluid and blood samples demonstrated CAZ-AVI concentration levels 20-fold greater than the minimum inhibitory concentration in the first 60 minutes postinfusion, providing evidence for the utility of CAZ-AVI in treating KPC–*Klebsiella pneumoniae* central nervous system infections.

Central nervous system (CNS) infections caused by carbapenem-resistant *Enterobacteriaceae* (CRE) are difficult to treat. Selecting an effective antibiotic regimen to overcome antimicrobial resistance while achieving and maintaining adequate CNS levels is particularly challenging. Moreover, clinical accounts treating CNS infections with newer antimicrobial agents are lacking, and data pertaining to their pharmacokinetic/pharmacodynamic (PK/PD) properties remains scarce. Carbapenem-hydrolyzing β-lactamases such as *Klebsiella pneumoniae* carbapenemase (KPC) confer resistance to most β-lactams, including carbapenems [1]. The gene encoding KPC production (*bla*_KPC_) has spread globally on mobile genetic elements, and KPC currently constitutes the most common carbapenemase in the United States [2].

Avibactam (AVI) is a non-β-lactam β-lactamase inhibitor (BLI) that inhibits Amber class A (including KPC), C, and some class D β-lactamases. The pairing of ceftazidime/avibactam (CAZ-AVI) has exhibited excellent in vitro activity against KPC-producing Enterobacteriaceae [3]. Clinically, CAZ-AVI demonstrated superior efficacy against these isolates when compared with other established treatment regimens [4].

However, clinical and/or laboratory human data regarding the penetration of CAZ-AVI into the CNS are not available. In a rabbit model, both ceftazidime and avibactam exhibited 38% cerebrospinal fluid (CSF) penetration and decreased CSF bacterial loads by a mean of 5.66 log10 cfu over 8 hours [5]. We report a case of postneurosurgical meningitis caused by KPC-producing *K. pneumoniae* (KPC*-Kp*) that was treated with CAZ-AVI. The unique challenge posed by this infection compelled us to measure the pharmacological properties of CAZ-AVI. Therapeutic drug monitoring (TDM) was employed to evaluate the adequacy of therapy and guide the correct CNS dosing of CAZ-AVI.

## CASE PRESENTATION

**Figure 1.**
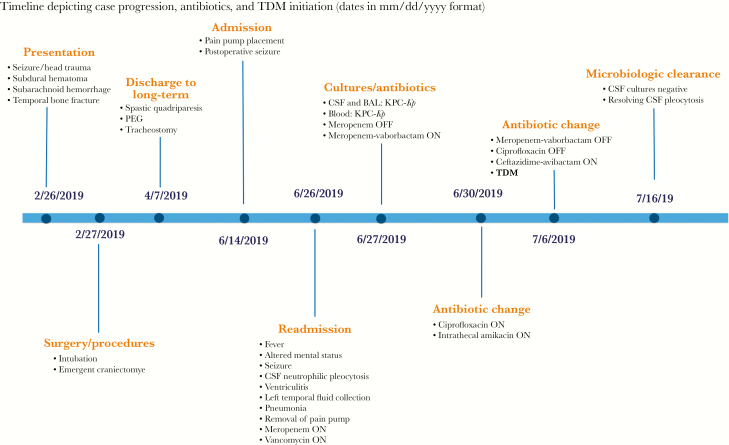
Clinical synopsis timeline. Abbreviations: BAL, brochoalveolar lavage; CSF, cerebrospinal fluid; KPC-*Kp*, *Klebsiella pneumoniae* carbapenemase–producing *Klebsiella pneumoniae*; PEG, percutaneous endoscopic gastrostomy; TDM, therapeutic drug monitoring.

A 38-year-old man with a history of alcohol dependence was transported to the emergency department following head trauma that resulted in a left temporal bone fracture and intracranial bleeding. The patient underwent urgent craniectomy followed by several burr hole neurosurgical evacuation procedures. His hospitalization course was complicated by prolonged mechanical ventilation, tracheostomy, and spastic quadriparesis necessitating the placement of an intrathecal pump. He was discharged to a long-term chronic care facility but was readmitted with delirium, seizures, dyspnea, and high-grade fevers (39.8°C). Computed tomography (CT) scan of the brain revealed diffusely inflamed ventricles and a left temporal fluid collection. CT scan of the chest exhibited focal opacification in the right upper and middle lung. Pertinent laboratory testing revealed leukocytosis (21 ×10^3^/mm^3^ with 86% neutrophils). A lumbar puncture was significant for CSF neutrophilic pleocytosis (white blood cell count 325/mm^3^, 94% polymorphonuclear cells), elevated CSF protein (>200 mg/dL), and low CSF glucose level (< 20 mg/dL) indicative of acute pyogenic meningitis. Blood, sputum, and CSF cultures were collected, after which the patient was treated empirically with meropenem and vancomycin. His intrathecal pump device was removed on the first day of admission. All collected cultures identified growth of *K. pneumoniae* with resistance to carbapenems, prompting a switch in therapy to meropenem-vaborbactam (MV). Transthoracic echocardiography (TTE) did not reveal the presence of any valvular vegetations. Drainage of temporal lobe abscess was subsequently performed as the patient became hemodynamically stable. His clinical course was thereafter marked by sustained ventriculitis, worsening neutrophilic pleocytosis, and persistent carbapenem-resistant *K. pneumoniae* (*CRKP*) bacteremia. Ciprofloxacin was consequently added to the treatment regimen (see timeline) as the patient developed hospital-acquired pneumonia. Follow-up susceptibility testing demonstrated MV and CAZ-AVI minimum inhibitory concentrations (MICs) of 4 µg/mL and 0.75 µg/mL, respectively. After 10 days of therapy, MV was replaced by CAZ-AVI and amikacin was administered intrathecally. Postinfusion TDM of CAZ-AVI CSF concentrations was performed to assess the adequacy of therapy and ensure sufficient CAZ-AVI levels in the CNS. CAZ-AVI was dosed at 2.5 g administered over 2 hours every 8 hours. All follow-up cultures were negative, and gradual clinical improvement ensued. A summary of the case synopsis timeline is shown in Figure 1.

## METHODS

Initial bacterial identification and antimicrobial susceptibility testing (AST) were initially conducted via the MicroScan System (Siemens Healthcare Diagnostics). Subsequent confirmation was performed using matrix-assisted laser desorption ionization (MALDI). Additional MICs were determined using broth microdilution and disc diffusion assays. Interpretation of AST results was based on Clinical and Laboratory Standards Institute (CLSI) guidelines [6]. Polymerase chain reaction (PCR) testing for common antimicrobial resistance genes including *bla*_KPC_, *bla*_NDM_, and *bla*_OXA48_ and whole-genome sequencing (WGS) were performed.

To protect patient anonymity and private health information, recognizable identifying information was not employed in this write-up. Additional procedures and testing were conducted in accordance with HIPAA regulations. The treatment plan was decided by the patient’s treating physician and was not altered as a result of our study approach. CSF was obtained via standard lumbar puncture (spinal tap) procedure. TDM was conducted using a CAZ-AVI dose of 2.5 g intravenously given as a 2-hour infusion every 8 hours. The first CSF sample was obtained after 3 completed CAZ-AVI doses specifically collected at 130 minutes after initiation of infusion. Follow-up CSF and blood samples (#2, #3, and #4) were thereafter collected to measure antibiotic concentrations beyond the passage of 1 avibactam half-life. These levels were obtained at 184 minutes after initiation of CAZ-AVI infusion. All samples were centrifuged and stored at –70°C until the time of interpretation. Quantification of serum and CSF concentrations was performed using a validated chromatography-tandem mass spectrometry assay (LC-MS/MS) developed by Keystone Bioanalytical (North Wales, PA, USA).

## RESULTS

*K. pneumoniae* was identified by bacterial subculture and confirmed by MALDI. AST revealed resistance to all penicillins, cephalosporins, carbapenems, aztreonam, and fluoroquinolones. The MICs of CAZ-AVI, MV, and amikacin were 0.75 μg/mL, 4 μg/mL, and 4 μg/mL respectively. Multilocus sequence typing (MLST) identified a non-ST-258 strain. PCR and WGS demonstrated the isolate to harbor the *bla*_KPC-3_ gene. *bla*_NDM_ and *bla*_OXA48_ alleles were not detected. In addition, WGS uncovered the presence of other resistant determinants including *fosA6*, *dfrA14*, *sul1*, *bla*_OXA-1_, *bla*_SHV-214_, *mphA*, *catB3*, *arr-3*, and numerous genes encoding aminoglycoside acetyltransferases and phosphotransferases. See Table 1B for complete WGS results. The measured serum and CSF concentrations of AVI and CAZ are displayed in Table 1A.

**Table 1.** Therapeutic Drug Monitoring of CAZ-AVI Concentrations and Whole-Genome Sequencing

A, Therapeutic Drug Monitoring of Ceftazidime-Avibactam Concentrations in CSF and Blood				
Specimen	Sample Collection Time, Minutes After Initiating Infusion		Concentration, μg/mL	
			Ceftazidime	Avibactam
CSF #1	130		19.007	4.242
CSF #2	184		17.27	3.917
CSF #3	184		17.244	4.099
CSF #4	184		19.727	4.148
Blood	184		61.273	13.085
B, Whole-Genome Sequencing of *KPC-Kp* Isolate				
Plasmid	Gene	Enzyme	Drug Class	% ID
108 kb	*sul1*	Sulfonamide-resistant dihydropteroate synthase	Sulfonamide	100
108 kb	*dfrA14*	Trimethoprim-resistant dihydrofolate reductase	diaminopyrimidine antibiotic	100
108 kb	*oqxA/B*	RND antibiotic efflux pump	fluoroquinolone tetracycline diaminopyrimidine glycylcycline, nitrofurans	100
160 kb (KPC+)	*aac(3)-Ib*	Aminoglycoside 3’-N-acetyltransferase	Aminoglycoside	100
160 kb (KPC+)	*aac(6’)-Ib-cr*	AAC(6’)-Ib-cr aminoglycoside acetyltransferase	Aminoglycoside	100
160 kb (KPC+)	*aph(3’)-Ia*	Aminoglycoside 3’-phosphotransferase	Aminoglycoside	100
160 kb (KPC+)	*aph(3’)-Ia*	Aminoglycoside 3’-phosphotransferase	Aminoglycoside	100
160 kb (KPC+)	*bla*_OXA-1_	OXA-1/OXA-30 β-lactamase	β-lactam	100
160 kb (KPC+)	*bla*_SHV-214_	SHV-214 β-lactamase	β-lactam	100
160 kb (KPC+)	*bla*_KPC-3_	*Klebsiella pneumoniae* carbapenemase-3	Class A carbapenemase	100
160 kb (KPC+)	*mrx*	Macrolide resistance protein	Macrolide	100
160 kb (KPC+)	*mphA*	Macrolide 2’-phosphotransferase I	Macrolide	99.67
160 kb (KPC+)	*qacH*	Multidrug efflux pump	Multidrug transporters and regulators	98.18
160 kb (KPC+)	*catB3*	Chloramphenicol acetyltransferase	Phenicol	100
160 kb (KPC+)	*arr-3*	Rifampin ADP-ribosyltransferase	Rifampicin	100
160 kb (KPC+)	*sul1*	Sulfonamide-resistant dihydropteroate synthase	Sulfonamide	100

Abbreviations: CAZ-AVI, ceftazidime/avibactam; CSF, cerebrospinal fluid; KPC, *Klebsiella pneumoniae* carbapenemase.

## DISCUSSION

A 38-year-old man who developed postneurosurgical meningitis and bacteremia with a KPC-3-producing multidrug-resistant (MDR) *K. pneumoniae* isolate was effectively treated with CAZ-AVI and intrathecal amikacin. This case brought forth a distinct challenge that required balancing in vitro activity with pharmacokinetics. The lack of therapeutic options given the highly resistant nature of this organism necessitated considering CAZ-AVI, a compound with undefined CNS pharmacokinetics. Fortunately, the inherent PK properties of AVI make it a relatively consistent agent to dose. In general, AVI is cleared renally with an average *t*_1/2_ of 160 minutes [7] and is neither significantly affected by peripheral tissue efflux transporters nor metabolized by hepatic CYP enzymes [8]. The principal PK components (volume of distribution, *t*_1/2_, renal excretion) of AVI are complementary to those known for CAZ, and both exhibit low plasma protein binding, which suggests that their free unbound fractions would be available for dissemination into the CNS. In fact, AVI has the lowest plasma protein binding (<10%) compared with all other BLIs [9]. Compared with vaborbactam (which the patient received initially), AVI demonstrates a higher unbound fraction and a longer *t*_1/2_. Moreover, AVI does not demonstrate significant antibacterial activity and therefore does not alter the PD of CAZ.

CAZ-AVI is currently approved for intra-abdominal infections, complicated urinary tract infections, hospital-acquired bacterial pneumonia, and ventilator-associated bacterial pneumonia. The clinical experience with CAZ-AVI in the treatment CNS infections is uncommonly reported, and the capacity of this combination to cross the blood–brain barrier remains unknown. In this setting, effective therapy depends on the ability of CAZ-AVI to maintain elevated CSF concentrations that achieve PK/PD thresholds seen in prior simulation models for CNS infections. The only available experimental data were conducted in an animal model inoculating AmpC-producing *K. pneumoniae* into healthy rabbits. The CAZ-AVI mean CSF penetration (defined as the area under concentration (AUC) vs time curve for serum and CSF [AUC_CSF/Serum_]) was determined to be 38%, while bacterial loads decreased by a mean of 5.66 log10 CFU as a result of therapy [5].

In our patient, TDM of CAZ and AVI was conducted to determine how effectively CAZ and AVI distributed into the CNS. Consistent with the Food and Drug Administration–approved dosing, the patient received 2.5 g of CAZ-AVI administered as a 2-hour infusion every 8 hours (ie, total daily dose of 7.5 g). Based on our current understanding of the target PK/PD indices for CAZ-AVI [9–11], the measured postdose concentrations suggest that adequate CAZ and AVI exposures were achieved in the CSF and plasma. At 184 minutes postinfusion after 3 days of therapy (11 completed doses), the concentrations of CAZ and AVI in CSF were 19.0 µg/mL and 4.2 µg/mL, respectively. Correspondingly, the concentrations of CAZ and AVI in plasma were 61.3 µg/mL and 13.1 µg/mL, respectively. When CAZ is paired with AVI, the PK/PD target associated with bacterial killing is estimated to be ~50% fT >MIC [12]. For this patient, conservatively assuming a 2-hour *t*_1/2_ for CAZ and AVI, concentrations were likely well above the MIC for the entire 8-hour dosing interval in the CSF and plasma. In fact, CAZ concentrations were likely 4–5 times higher than the MIC for the entire dosing interval in both the CSF and plasma [12]. Similarly, the PK/PD index for AVI is estimated to achieve concentrations ≥1–2.5 mg/L for ~50% of the dosing interval (% time above a threshold) [9, 13] and this was likely attained at both sites. Unforunately, clinical challenges precluded obtaining additional CSF samples to estimate the actual concentration-time profile in the CSF. Ideally, the true drug elimination rate constant could be calculated using ~2–3 supplementay samples collected a few hours apart during the same dosing interval or at different times across 2 different dosing intervals. However, based on the current understanding of CAZ-AVI critical PK/PD indices, we ascertain that the conservatively extrapolated exposure profiles of CAZ-AVI using the population estimates of their elimination rate constants concentrations were likely sufficient. Moreover, the CSF CAZ-AVI concentrations and PK findings from this study are likely understated, as samples were collected at a time of relatively reduced meningeal inflammation (ie, after 9 days of therapy and infection onset). Meningeal inflammation is known to improve the penetration of β-lactam antibiotics into the CSF [14, 15]. Therefore, it is reasonable to conclude that the CSF PK findings from this study are a conservative estimate of CAZ-AVI CSF penetration, but further studies are needed to verify these measurments.

Only 2 published reports have described CRE-associated CNS infections treated with CAZ-AVI. In both cases, postneurosurgical meningitis was caused by CRKP (KPC production was not confirmed). Samuel et al. [16] employed CAZ-AVI monotherapy dosed at 2.5 g every 6 hours, while Holyk et al. [17] treated their patient with a combination of CAZ-AVI and intraventricular amikacin. Direct injection of antibiotics into the CNS is indicated with worsening infection or persistent pleocytosis. Intrathecal amikacin was therefore added to our patient’s therapy, albeit with an observed MIC at the breakpoint for this isolate. The optimal duration of treatment for a postneurosurgical CNS infection with KPC-*Kp* is unclear. Our patient received an additional 2 weeks of CAZ-AVI and intrathecal amikacin to achieve resolution of CSF neutrophilic pleocytosis and microbiologic cure. Although the objective of measuring CAZ-AVI concentrations in the CSF of a patient with a KPC-*Kp*-associated CNS infection was achieved, these data should be interpreted with caution as they represent only 2 CSF sample measurements. Furthermore, conclusions regarding CAZ-AVI serum ratios/percentages cannot be generalized using this study design due to the sample size, interpatient variability, and other clinical variables.

Additional studies that measure CSF concentrations of CAZ-AVI using more samples from multiple patients and throughout the entire dosing interval are required to determine the PK/PD parameters for CNS infections with highly resistant organisms. However, the levels attained and measured in our patient using this dosing regimen appear to have been adequate.

## CONCLUSIONS

This report describes the measurement of CAZ-AVI concentration levels in the CSF of a patient suffering from *KPC-Kp* nosocomial meningitis. A novel CAZ-AVI TDM method was successfully applied to demonstrate that CAZ-AVI achieves CSF levels well above the MIC in the first 180 minutes postinfusion. Further clinical studies are required to assess the complete profile of CAZ-AVI in the CSF. Our case provides evidence on the use of this agent in treating KPC-*Kp*.
